# New products

**DOI:** 10.1155/S1463924690000165

**Published:** 1990


Journal of Automatic Chemistry, Vol. 12, No. 3 (May-June 1990), pp. 129-140

### New products

### Portable ppm $O_2$ analyser

Teledyne's TURBO2 is a compact trace oxygen ($O_2$) analyser that provides parts-per-million (ppm) sensitivity and true portability. Weighing just 7 lb, the TURBO2 quickly and simply spot-checks gas phase ppm $O_2$ content of a variety of industrial gasses and gas mixtures.

The TURBO2 uses Teledyne's patented Class S-2 Micro-Fuel Cell $O_2$ sensor. response with the S-2 sensor is fast: 90% of full scale in 5-10 s. With the S-2, no zero gases or special span gases are required—air (209, 500 ppm $O_2$) is used for calibration; and the S-2 recovers quickly from exposure to air, allowing accurate low-ppm $O_2$ measurements in less than 30 min after calibration.

The TURBO2 features integral rechargeable batteries which will power the analyser for up to 30 days between charges. The TURBO2 also features three measuring ranges of 0-100/0-1000/0-10 000 ppm $O_2$, a special span range for air calibration, quick-disconnect gas line fittings, and an easy-to-read analogue meter.

*Details from Teledyne Analytical Instruments, The Harlequin Centre, Southall Lane, Southall, Middlesex UB2 5NH, UK. Tel.: 081 571 9596; fax: 081 571 9439.*

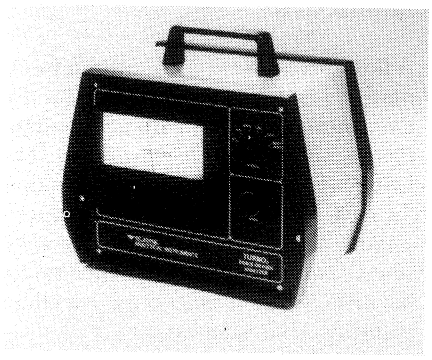

*Teledyne's portable oxygen analyser which measures ppm concentrations in 5 to 10 seconds.*

### Particle-free sampling

The Biopem aseptic continuously-operating sampling device from the Biotechnology Division of B. Braun Medical Ltd is extensively used for on-line measurement of biochemical reactions in fermentation. However, with growing concern being expressed over water quality in rivers and the effectiveness of water treatment, Biopem is gaining increasing acceptance for monitoring in these areas.

Biopem is a magnetically-stirred filtration cell which provides a continuous and representative flow of particle-free samples, with short and reproducible response times. Robustly constructed in stainless steel, the device utilizes a variety of 90 mm diameter microfiltration and ultrafiltration membranes. By effec-

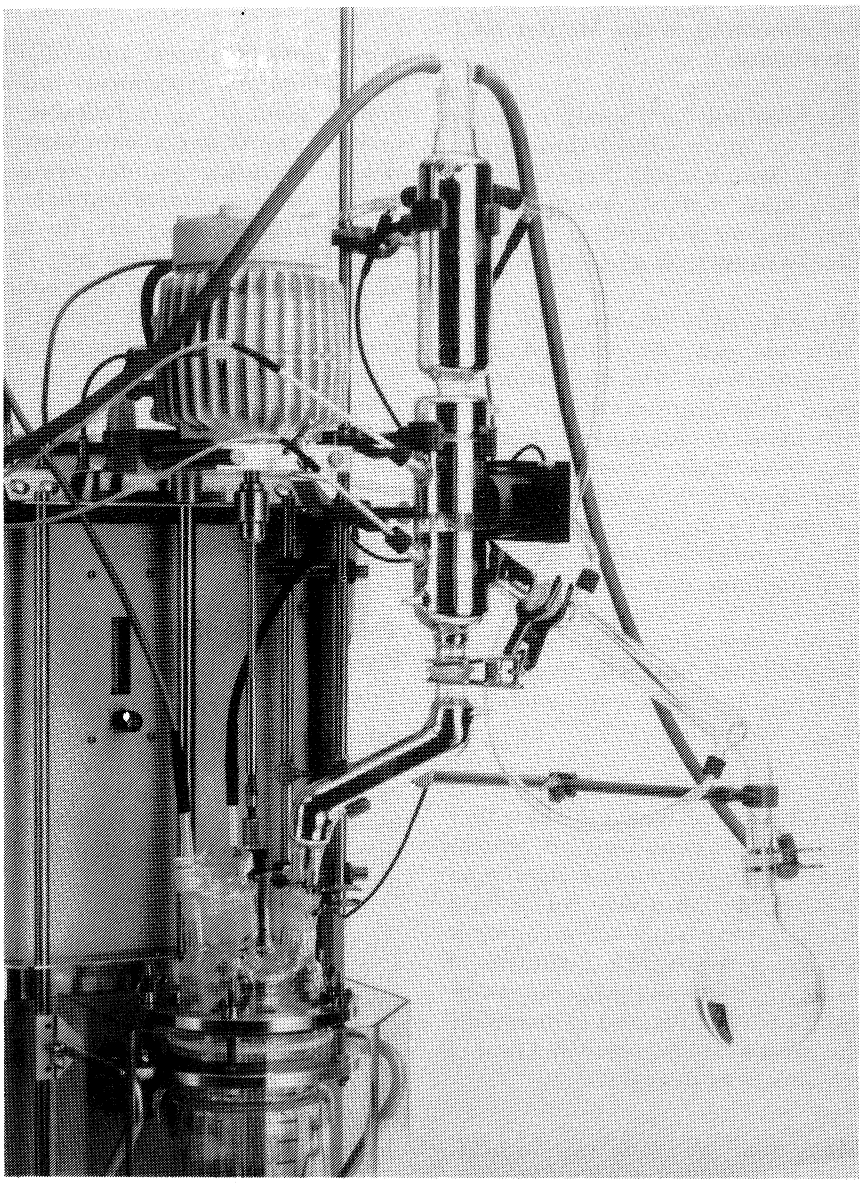

*The updated Mettler RC1 laboratory reactor—correct heat balancing is achieved at boiling point, in distillation and in refluxing.*

tive control of flow-rate, stirrer speed and stirrer height, Biopem yields excellent long-term stability with minimal membrane fouling.

The unit can be interfaced with any liquid analysis system and, when used in conjunction with continuous on-line monitoring systems, it is a highly-effective tool for river monitoring and water treatment monitoring.

*More information from B. Braun Medical Ltd, Braun House, 13–14 Farmbrough Close, Aylesbury Vale Industrial Park, Stocklake, Aylesbury, Buckinghamshire HP20 1DQ, UK. Tel.: 0296 393900; fax: 0296 435714.*

### Enhancement of the Mettler RC1 lab reactor

The Mettler RC1 laboratory reactor has been improved with the addition of the DEST 29/32 Refluxing and Distillation Set accessory: correct heat flow measurement is now possible in distillation and refluxing.

The laboratory reactor with heat balancing can be controlled by a personal computer and allows acquisition of accurate calorimetric data on chemical reactions and phase transitions under conditions resembling those in the plant. These data are used by chemical engineers as a basis for the development of economically optimized and safe industrial processes. All components of the flexibly expandable system are so designed that the results obtained on a litre scale can be extrapolated to plant conditions.

The calorimetric information is obtained by balancing the heat flow through the double-walled reactor jacket. This contains a circulating heat transfer oil which can be used for the rapid dissipation or supply of accurately measurable quantities of heat. An evaluation program can be subsequently employed to determine the exact heat flow curve and heat of reaction from the data.

Many unit operations, such as heating, cooling, dispensing or stirring, can be controlled through regular manual entry or stored action programs. In the temperature range −20 to 200 °C, isothermal, isoperibolic or adiabatic temperature control is possible. Temperature, pressure and pH values, as well as other user-specified measured values, can be used as reference inputs for process controllers. All values are continuously displayed on the screen and can be recorded in curve form.

Many chemical reactions are run at ambient pressure and at the maximum possible temperature under reflux conditions. Such conditions allow achievement of a high rate of reaction and – in the case of highly exothermic reactions – efficient cooling. The heat flow due to the vaporization of solvents or reactants is usually large.

For reasons of process authenticity, with laboratory experiments run at boiling point, it is undesirable or even impossible to prevent vaporization by increasing the total pressure. In such cases, a correct heat balancing is made possible by the new Refluxing and Distillation Set. This allows determination of the cooling power of the reflux or distillation condenser. An electromagnetically operated swing-out funnel in the reflux divider enables work to be performed under either reflux or distillation conditions.

*Details from Mettler-Toledo AG, CH-8606 Greifensee, Switzerland.*

### Thin-layer chromatography catalogue

*TLC 90* is CAMAG's new, 44-page catalogue which covers all aspects of thin-layer chromatography. The catalogue gives detailed product information, as well as information about TLC techniques and methodology.

An introductory section outlines the individual steps of thin-layer chromatography and specifies the instruments required. Then the individual instruments are presented in their order of use: sample application, chromatogram development and densitometric evaluation. Instruments for special techniques, such as post-chromatographic derivatization, UV application and photodocumentation, then follow and, finally, instruments for in-house preparation of TLC plates. The consumables required are also mentioned.

The catalogue provides a list of TLC analysis methods obtainable from CAMAG, classified by fields of application; in addition there are references to literature, audiovisual teaching aids and a key-word index.

*Copies from CAMAG, Sonnenmattstrasse 11, CH-4312 Muttenz, Switzerland. Tel.: 061 61 34 34; fax: 061 61 07 02.*

### Custom report formatting software

New report formatting utility software for 700 Series chromatography workstations allows users to easily create customized reports from standard data system result files. Reports can include intra- or inter-run statistics and tabulations of selected peaks from multiple injections. This software also includes batch file translation utilities which can quickly convert existing chromatogram and report files to various WKX formats directly compatible with versions of Lotus 1-2-3.

The report formatting software is simple to install and run on any Axxiom Chromatography 727, 737, 747, or remote editor system.

*Note new Axxiom Chromatography address: 11988 Challenger Court, Moorpark, California 93021-7122, USA. Tel.: 805 523 8888; fax: 805 523 7900.*

### New LS system detects low-level radioactivity

A liquid scintillation counter in Beckman's LS 6000 Series is ideal for environmental and other low-level radioactivity sample counting. The LS6000LL offers high performance for single, dual or triple label experiments. The instrument utilizes solid scintillation media that reduce safety hazards, while maintaining excellent counting characteristics.

The LS6000LL features Beckman's H-number Plus and Xtalscint, CPM/DPM, luminescence correction, single and dual label DPM, two-phase monitor and auto-DPM.

The patented H-Number Plus offers the environmental laboratory researcher the improved statistical accuracy of an external quench monitor, with multiple-label experiments now more accurate over a wider quench range. New features on the LS6000 Series include colour correction which automatically corrects for sample colour, luminescence correction, Xtalscint CPM/DPM which provides disintegrations-per-minute for solid scintillator samples, and auto-DPM which performs accurate single label DPM measurements of any pure beta or alpha emitting isotope without the need for quench curves.

An 80-column printer is included, together with customized print-outs, a complete set of standards or miniature racks, alphanumeric keypad, high activity monitor, user accessible diagnostics, automatic calibration with verification, electrostatic controller, help screens, isotope library, Auto-Isoset automatic background subtract, sample identification, half-life correction, normalization factors, sample repeat and replicate averaging with coefficient of variation calculations, permanent memory and an interrupt mode.

*Details from Beckman, Progress Road, Sands Industrial Estate, High Wycombe, Buckinghamshire, UK. Tel.: 0494 441181.*

### Photodiode array spectrophotometers

Hakuto International UK Ltd are distributing the innovative range of Otsuka Electronics Photodiode Array Spectrophotometer systems. Two models are offered, the MCPD-1000 and the IMUC-7000 intensified system, for low light level measurements. Both systems use fibre optic technology for the input of spectra.

All models incorporate accurate 16 bit A/D convertors and 512 channel photodiode arrays, which can be scanned in only 16 ms, enabling the observation of fast transient phenomena in the UV to near IR (220–1100 nm) regions.

Comprehensive software for IBM PC/AT and compatibles, provided as standard, includes routines for wavelength calibration, auto and manual measurement of multiple spectra, data correction and inter-spectra operations. Additionally, a variety of optional software routines and hardware is available, enabling the system to be customized to meet the requirements of specific applications.

The versatility of the systems ensures that a wide range of applications, covering both absorption and emission techniques, can be addressed. Typical of these are: emission analysis of plasma, chemical and bio-luminescence, characterization of semiconductor materials and light sources, absorption analysis of fast chemical reactions, thickness measurement of thin films and *in vivo* spectroscopy.

*More information from Hakuto International UK Ltd, 33–35 Eleanor Cross Road, Waltham Cross, Hertfordshire EN8 7LF, UK. Tel.: 0992 769090.*

### High-quality CI Spectra

The analytical power of Hewlett-Packard's low-cost HP 5971A mass selective detector (MSD) has been increased by the introduction of a chemical ionization (CI) accessory. With this, the MSD provides molecular weight information and increased sensitivity and selectivity for many compounds. CI is very useful when a compound cannot be identified through a library search matching of its electron impact (EI) spectrum.

The HP 5971A is now the only MSD system which produces true, classical CI spectra. Chemical ionization is a soft ionization technique producing spectra with an enhanced molecular ion, which indicates the molecular weight of the compound. For many compounds, especially straight chain alkanes, alkenes and alcohols, this information is difficult to obtain from EI spectra. As CI yields just a few ions of relatively high abundance and at a relatively high mass, it can increase both sensitivity and selectivity for such compounds.

The new accessory comprises a separate ion source optimized for CI; modifications to the MSD interface; a reagent gas flow controller; and the required plumbing. After initial installation, the CI mode is effected by changing the ion source and introducing reagent gas under controlled pressure. Software macros are provided for automatic tuning with methane reagent gas and PFTBA calibration compound.

Despite the compact size of the MSD (18 cm wide), excellent CI conditions are achieved. The high quality classical CI spectra obtained can be compared with reference spectra and results from other mass spectrometers. The accessory operates in positive CI mode, and is designed for use with narrow bore capillary GC columns. A variety of reagent gases may be used including methane, ammonia, isobutane and hydrogen.

*Enquiries to: Verena Haller, Hewlett-Packard SA, 150 Route du Nant-d'Avril, CH-1217 Meyrin (GE) 2, Switzerland.*

### New graphics power for HP Lab automation systems

New graphics power has been added to HP 3350A and HP 3359A laboratory automation systems (LAS) with the introduction of the HP 19471A advanced graphic chromatogram processor. This allows chromatograms to be displayed and compared, chromatographic data re-processed graphically, and publication quality reports produced.

*Details from Verena Haller at HP (as above).*

### Pumps for the water industry

Hanna Instruments' Model DP 7916 has a unique proportional cycle that operates when the measurement reaches 1·5 pH and ensures extremely accurate dosage. With the difference between actual value and the working level exceeding 1·5 pH the pump doses continuously, but below 1·5 pH the pump stops for a period proportional to the difference. This ensures that it is impossible to overdose.

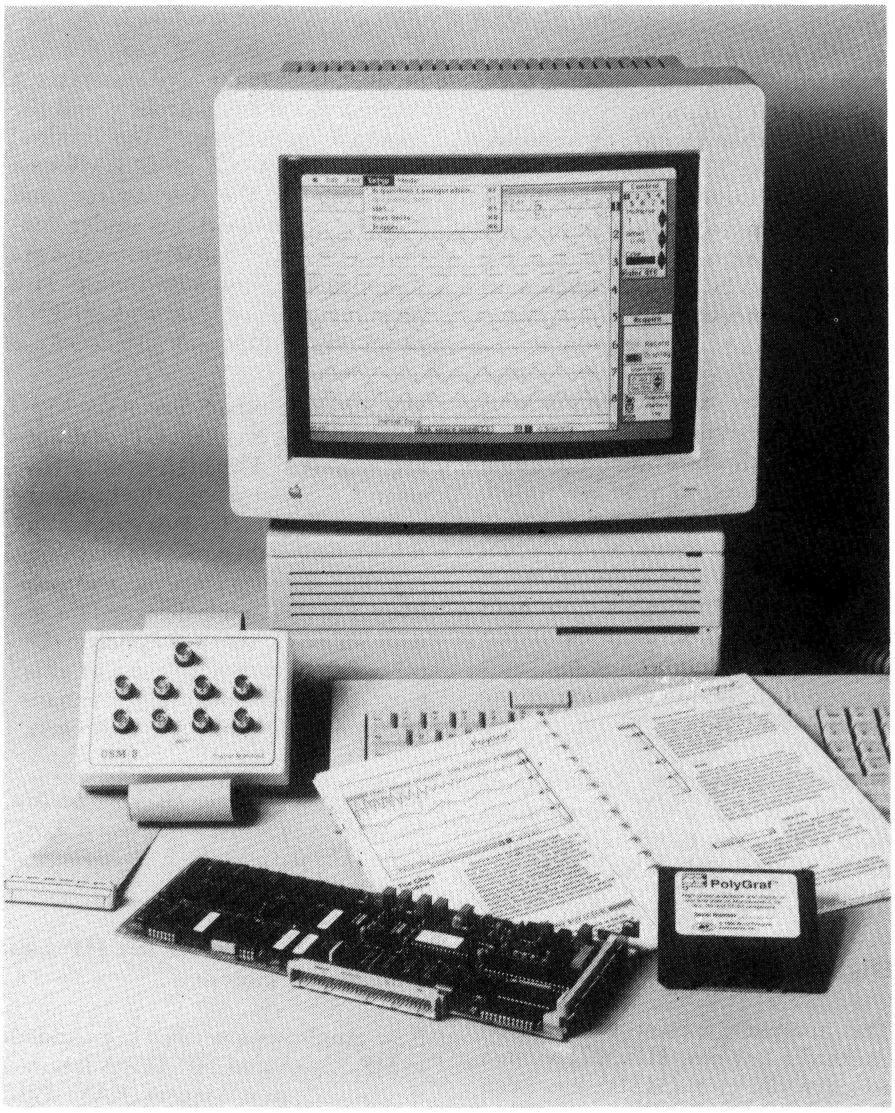

*The PolyGraf/8 system is a combined hardware and software package for the acquisition and display of electronic data with Macintosh II computers. Hardware consists of the PolyGraf/8 data acquisition card and the SM/8 Signal Manifold which provides a TTL-compatible trigger input and transports up to eight channels of raw data to the PolyGraf/8 card for a maximum acquisition speed of 1000 samples per second for each of the eight channels – up to 8000 data samples being collected, displayed, and saved every second with no gaps in the collected data. Recording and examining the acquired information is made possible through PolyGraf software which blends features found in data loggers, chart recorders and VCRs to provide a quick, powerful and intuitive user interface designed for 'plug and play' operation. Polygraf data files are fully compatible with WavEdit, a waveform editing program released by WPI earlier this year, permitting detailed manipulation and analysis of the acquired data. For more information contact World Precision Instruments, 375 Quinnipiac Avenue, New Haven, Connecticut 06513, USA. Tel.: 203 469 8281.*

DP7916 can control and regulate discharges of cyanide, chrome or high volume neutralization and is suitable for hydrochloric and sulphuric acid, caustic soda, sodium hypochlorite, sodium bisulphite, plus other acid or alkaline products.

The DP7916 is part of a range which includes pumps for adjusting pH in swimming pools and drinking water purification, an ORP controller, hospital and biological laboratory discharges, cooling towers and air conditioning and refrigeration plants.

All Hanna pumps are housed in a tough aluminium alloy casing with polypropylene pumpblock mounted on a stainless steel plate. Electrics are protected by a step-down transformer, automatic pressure release valve, and thermostatic circuit protection. Units are easy to install with components that come into contact with liquids supplied in materials resistant to chemical solutions.

*Details from Hanna Instruments Ltd, Happy Valley Industrial Park, Primrose Hill, Kings Langley, Hertfordshire WD4 8HZ, UK. Tel.: 09277 60655.*

### Micropipette puller

Research Instruments' new combination of versatile micropipette puller and novel plug-board power controller facilitates the reproducible production of micropipettes and microelectrodes in a wide range of shapes and sizes.

A number of pulling techniques may be employed, some of which achieve both first and second pulls on the same instrument.

Essentially, the MPP11 micropipette puller consists of two extremely lightweight carriages fitted with rapid-action clamps for holding the glass tube to be pulled, an adjustable-force pulling mechanism, and a heating filament in an interchangeable holder.

Preset outputs on the PCP6 plug-board power controller avoid the need for continual adjustment of the filament power when making two-pull pipettes, or while using the microforge – not only does this make operation much more rapid, but it also greatly reduces the chance of accidental errors.

The PCP6 has four presettable outputs for heating filaments, two for the micropipette puller and two for the RI microforges. Also provided are two 15W 6Vac presettable outputs for illuminators.

In addition to the MPP11 micropipette puller, RI make a full range of micromanipulators and micropipette preparation equipment including a new microbeveller. The micromanipulators are available in a comprehensive range to fit directly onto all large inverted microscopes, or onto cast bases for other types of microscope.

*More information from Research Instruments Ltd, Kernick Road, Penryn, Cornwall TR10 9DQ, UK. Tel.: 0326 72753.*

### Stable calibration gas mixtures

BOC Ltd has launched a new range of stability-assured gas mixtures for sensitive instrument calibration in such areas as occupational health, environmental monitoring, automotive engineering, the petrochemical industry and chemical processing. Designated Spectra-K, the mixtures are formulated for calibration in the parts per million and parts per billion ranges and are stability-guaranteed against drift for two years. The maximum deviation on a 1000 ppm mixture, for example, would be less than 1% over the two year period.

Analytical instrumentation is only as good as the gas standards used for calibration, and stability is of vital importance. Through the Control of Substances Hazardous to Health (COSHH) Regulations, regular sampling and analysis of the atmosphere in factories where a toxic substance is likely to be present is now a legal requirement. Spectra-K mixtures are ideal for calibrating the analytical instruments required.

Sensitive environmental monitoring equipment must be supported by accurate calibration and again Spectra-K is well-suited. The range covers binary mixtures of specific pollutants and multi-component mixtures for more general applications.

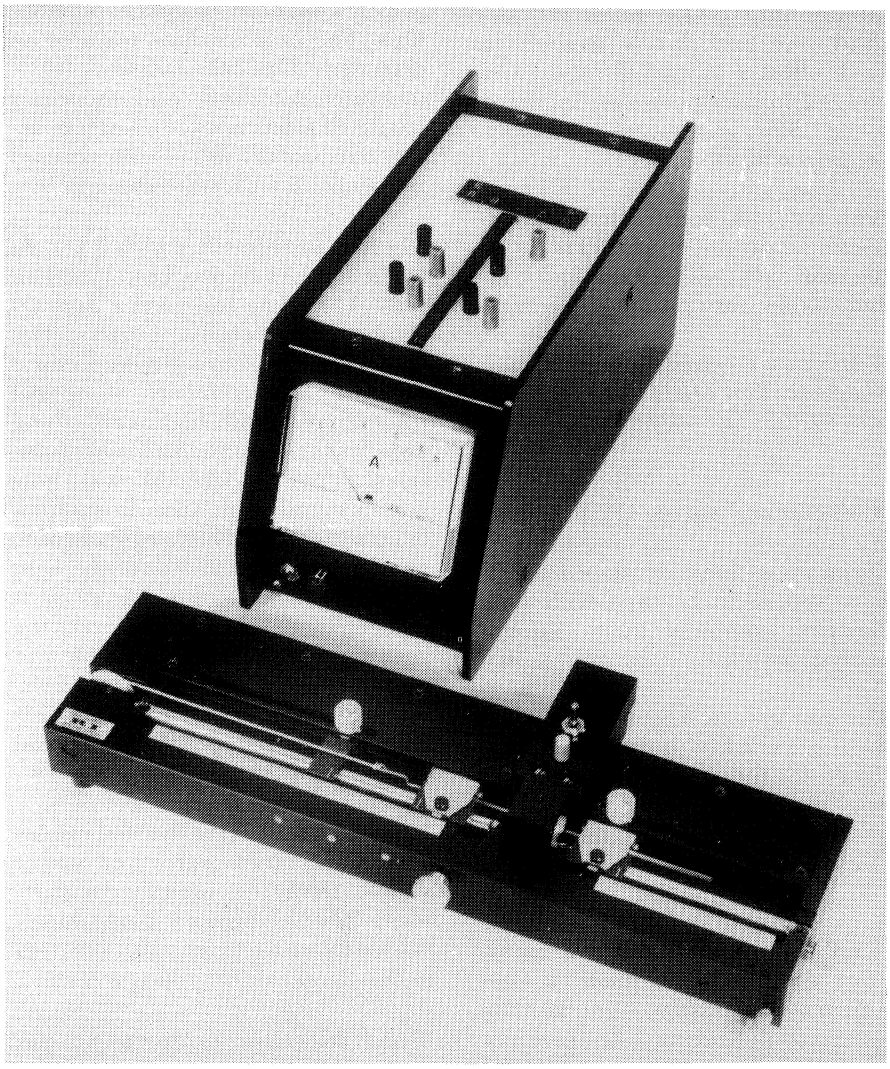

*Compact micropipette puller and plug-board power controller.*

The automotive industry requires stable gas standards for calibration of instruments for exhaust emission tests, evaporative loss testing, field surveillance and engine performance and efficiency.

In the petrochemical and chemical processing industries, calibration mixtures are required to calibrate process analysers – on-line instruments that monitor critical components and impurities. The low-level binary mixtures in the Spectra-K range come into their own in this application.

To qualify for registration under BS 5750, companies will need to demonstrate quality control against guaranteed standards. A typical application for Spectra-K in this area is for process control.

BOC's Spectra-K process comprises cylinder preparation and filling followed by analysis. Cylinder preparation uses a proprietary process designed to give a non-contributing surface to the interior of the cylinder. The components of the gas mixture are specified and analysed to ensure that they will not compromise the cylinder treatment. Component concentrations are analysed after filling and again one week later. These measurements must agree within the accuracy of the analytical method – normally ± 2 %.

The traceability of Spectra-K mixtures is an option which offers a direct traceable link between the calibration gas mixture and international standard reference material (SRM), combined with a stability guarantee.

*Details from BOC Ltd, The Priestley Centre, 10 Priestley Road, The Surrey Research Park, Guildford, Surrey GU2 5XY, UK. Tel.: 0483 579857.*

### Flexible data acquisition system

The Monitor flexible data acquisition system for both segmented flow and flow injection analysis can simultaneously process up to four systems or 16 channels of analogue signal.

The Monitor system has been designed for use in laboratory research

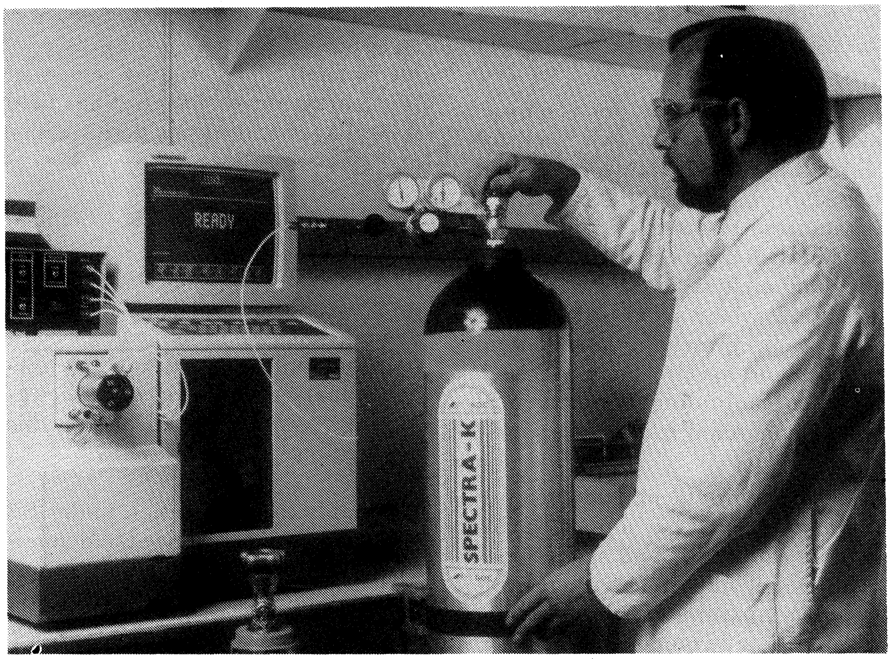

*Gas chromatograph calibration using a Spectra-K stability-assured gas standard.*

into water, soil and full liquid analysis with applications in water and waste monitoring, medical analysis, environmental control and industrial research.

For use with IBM and compatible PCs, the system can be linked to other standard database and spreadsheet software. The system is easy to use, with screen and user prompts.

Monitor will link to both other manufacturers' systems and Burkards own SFA-2 segmented flow system and flow injection analyser.

*More information from David Stelling, Burkard Scientific Ltd, PO Box 55, Uxbridge, Middlesex UB8 2RT, UK. Tel.: 0895 30056; fax: 0895 30058.*

### Disposable microfiltration device for aqueous-based HPLC solvents

Whatman Scientific Ltd has extended its range of microfiltration devices with a new disposable Aqueous In-Line Filter/Degasser (IFD). Designed to complement the Solvent IFD device, the new Aqueous IFD is used to simultaneously filter and degas aqueous HPLC mobile phases.

Both the IFD products consist of a shallow, cylindrical polypropylene housing that connects easily and directly into HPLC lines, via supplied nut and ferrule assemblies. Each offers a 16 $cm^2$ filtration area and 0·2 micron pore size; by use of nylon filter in aqueous and polypropylene in solvent IFD.

Both types are constructed to incorporate an air outlet vent. This is on the inlet side (with a Luer lock cap) and enables easy priming.

*Details from Whatman, Springfield Mill, Maidstone, Kent ME14 2LE, UK. Tel.: 0622 692022; fax: 0622 691425.*

### 'Can cracker' solves QC problem

Spectramass have developed a new inlet system to be used with their Compass computer integrated gas analysis system.

The new system, known in-house as the 'Can cracker' is specifically designed for the analysis of entrapped gases in such samples as semiconductors, pieces of glass or metals and electric light bulbs.

A customer-configured sample chamber is connected via a multivalve manifold to the high vacuum system of the mass spectrometer. The test piece is held on a temperature controlled sample stage and after the chamber has been evacuated down to a suitable level, it is broken open by a vacuum tight pointed plunger. Escaping gas can be monitored directly into the mass spectrometer through a precision leak valve or can be expanded into a reservoir for a more detailed look. Sample chambers are designed with quick release fittings to permit relatively rapid sample throughput and much useful information can be gained which enables more accurate quality control and process optimization.

*For more information contact Spectramass Ltd, Radnor Park Industrial Estate, Congleton, Cheshire CW12 4XR, UK. Tel.: 0260 279531.*

### Polarimeters

Optical Activity Ltd have launched the first instrument in a new family of Polarimeters which offer users greatly enhanced analytical possibilities. The new machine features an extended Optical Density (OD) measurement range and thermally insulated electronics and lamp housing to reduce the risk of errors caused by sample temperature changes.

The new instrument, which is known as the PolAAr 20, has been based on the AA10 range and gives a number of important benefits to users. The first of these is the ability to produce accurate measurements of optical rotation even with highly absorbing samples such as unrefined sugar solutions. The PolAAr 20 will now measure optical rotation in samples of up to 3·0 OD. This is equivalent to an absorbance of 99·9% of the light from the source lamp. A 20 watt halogen lamp has been chosen as the light source because it is reliable and gives excellent life – up to 2000 working hours in normal use. This will help to reduce downtime and routine maintenance costs.

Another feature is the thermal insulation of the lamp source and power supply from the sample compartment to eliminate inaccuracies caused by small temperature changes in the sample during measurement.

Switch selectable measurements can be made in either angular rotation (−90° to +90°) or International

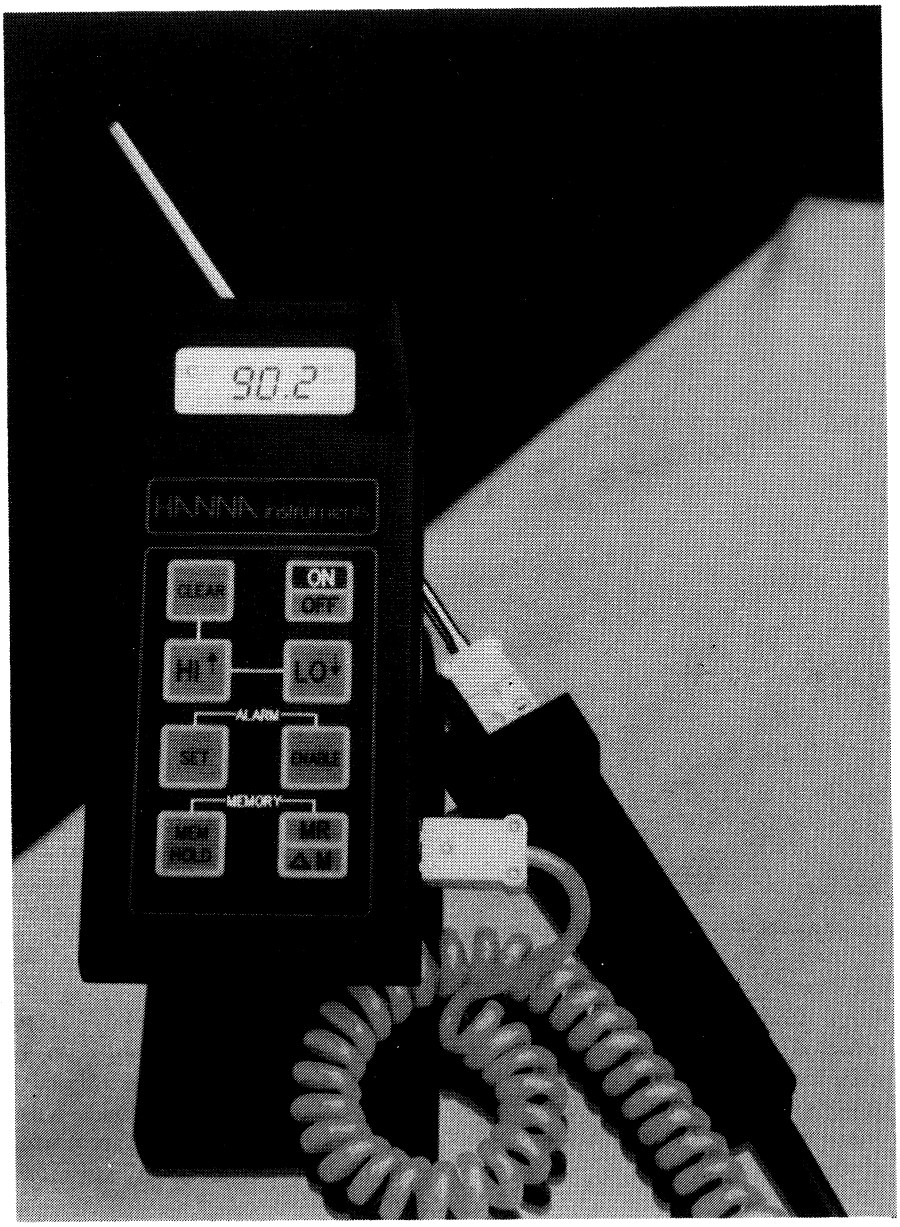

*A new Type K thermocouple electronic thermometer model HI 9053 is a portable, battery-powered instrument with a membrane keypad, and features microprocessor based electronics to compensate for drift in measurement circuits and to correct the linearity of the temperature sensing probe. Functions include storage of the highest and lowest reading during a continuous process, allowing the operator to check if temperatures have exceeded predetermined levels. Where it is critical for temperatures to remain at certain levels, high and low temperature limits can also be pre-set. Exceeding these limits will activate an audible alarm. The last instrument reading can also be held on the display by using the 'MEM/HOLD' key so that readings can be taken in difficult-to-reach locations and read later. Model HI 9053 has a switchable measurement range of −50 to +150°C with 0·1°C resolution, and −50 to +900°C with 1°C resolution. Further information from Hanna Instruments Ltd, Happy Valley Industrial Park, Primrose Hill, Kings Langley, Hertfordshire WD4 8HZ, UK.*

Sugar Scale units (°Z), and are displayed on a liquid crystal display. The PolAAr 20 also has an RS232 interface allowing it to be connected to a very wide range of automatic data acquisition equipment. This opens up many opportunities for routine and continuous on-line analysis functions, especially in process and quality control situations.

The PolAAr 20 has been designed to accommodate the full range of internationally standard sample cells from 5 mm to 200 mm pathlength, with flow-through and constant temperature control options as well.

*Further information on the new PolAAr 20 Automatic Polarimeter or applications advice is available from Optical Activity Ltd, Bury Road Industrial Estate, Ramsey, Huntingdon, Cambridgeshire PE17 1NA, UK. Tel.: 0487 813913.*

### Conductivity cell with built-in temperature sensor

Conductivity measurements are very temperature dependent. Consequently, in order to ease the analysis considerably, the new CDC04T cell from Radiometer Analytical is designed to get simultaneously measurements of conductivity and temperature.

The new cell is based on the proved CDC304 type having a bell-design with one inner and two outer electrodes, which secure a well-defined and stable cell constant. Incorporated in the new design is the temperature sensor at the same position as the measuring electrodes, so conductivity and temperature are correctly associated with each other and any automatic temperature correction is done in an optimal way.

The CDC304T is primarily used together with the CDM83 autoranging and autocalibrating conductivity meter. This meter has seven measuring ranges from 0·001 μS/cm to 1300 mS/cm. Range and frequency selection is done automatically using four different frequencies from 73 Hz to 50 kHz. Therefore, measurements at high conductivities are possible using cells with bright platinum electrodes.

*For further information, please contact Radiometer Analytical A/S, Krogshojvej 49, DK 2800 Bagsvaerd, Denmark. Tel.: 45 31696311; fax: 45 44490011.*

### Chromacol opens US office and warehouse

Chromacol, Europe's largest supplier of vials, caps and seals for

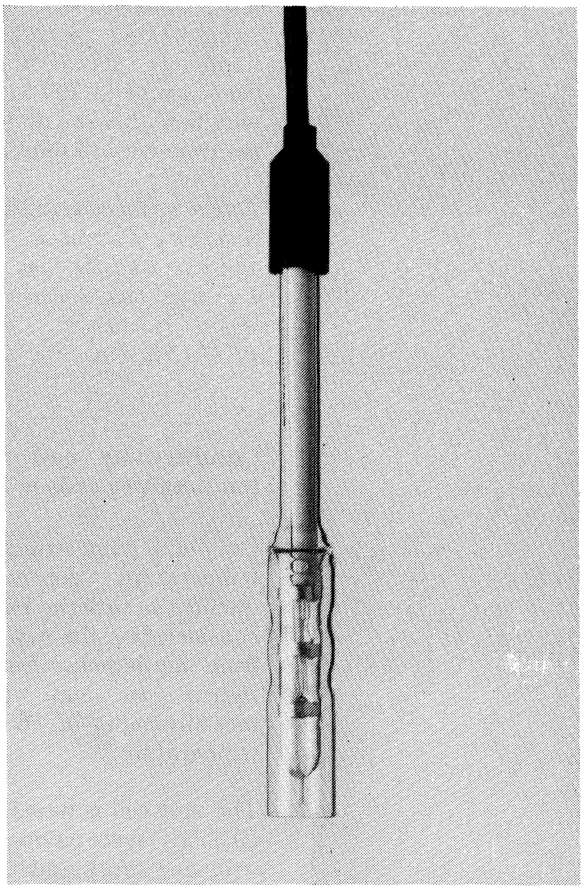

*The CDM83 has a free selectable reference temperature and may be connected to a printer, a computer, or a titration system.*

chromatography autosamplers, has opened an office and warehouse in Trumbull, Connecticut to service its rapidly growing business in the US. The address of the new office is Chromacol, PO Box 293, Trumbull, Connecticut 0661, USA.

Michele Slade will be President of the US operation and Andrew J. Baxter will be Vice-President, Marketing.

A major part of the new company's business is expected to come from chromatography instrument operators looking for rapid service, good technical support and a wide range of innovative products, benefits currently enjoyed by Chromacol's UK and world-wide customers.

*Chromacol's headquarters are at Glen Ross House, Summers Row, London N12 0LD, UK.*

### pH Measurement in pure water samples pH measurements are typically made in solutions which contain relatively large amounts of acid or base, or which contain substantial amounts of dissolved salts. Under these conditions, conventional pH electrodes make measurements quickly and precisely.

There has been growing interest in making pH measurements in 'pure water': water in which the total amount of acid or base is very small, and in which there is a low level of dissolved salts. The terms 'pure water' and 'low ionic strength' can be used interchangeably. Samples which may fall into this category include:

- Distilled water
- Deionized water
- Some process water
- Well water
- Some surface water
- Treated effluent
- Boiler feedwater
- Rain water

Measurement in these pure water samples is more difficult. Although electrodes respond quickly in buffers, in pure water the electrode response is unsatisfactory – slow, noisy, drifty, non-reproducible, and inaccurate.

Orion has developed a new method which minimizes the problems encountered when measuring pH in pure waters. The method uses a good quality glass pH electrode, a kit consisting of a pure water pH additive called pHiX adjustor (pronounced 'fix'), and a special set of diluted buffers containing the same background of pHiX adjustor. For best results, the ROSS Electrode, Model 8102 is essential.

*For full details of the Orion approach to pure water measurement, contact Orion Research UK, Freshfield House, Lewes Road, Forest Row, East Sussex RH18 5ES, UK. Tel.: 0342 824033.*

### Mains current irregularities

A range of uninterruptible power supply (UPS) systems has been introduced to protect computer systems, voice and data communications equipment, and plant room operations from any AC mains power supply disturbance or complete power failure.

Datapower static on-line UPS systems are based on advanced technology and it is claimed they will handle 100% computer loads without derating the UPS or requiring additional filters to deal with non linear loads.

Microprocessor controlled, the units are designed for compatibility with all existing computer systems, including IBM AS400 models. A remote control and supervision module for operation via an RS232 interface is available.

Datapower UPS systems are available from Oakwood Computer Services, which has also developed a fully automatic standby power supply package for telecommunications systems, incorporating the Datapower units.

*Details from Oakwood Computer Services Ltd, Commercial Avenue, Stanley Green Trading Estate, Cheadle Hulme, Stockport, Cheshire SK8 6QH, UK. Tel.: 061 488 4343.*

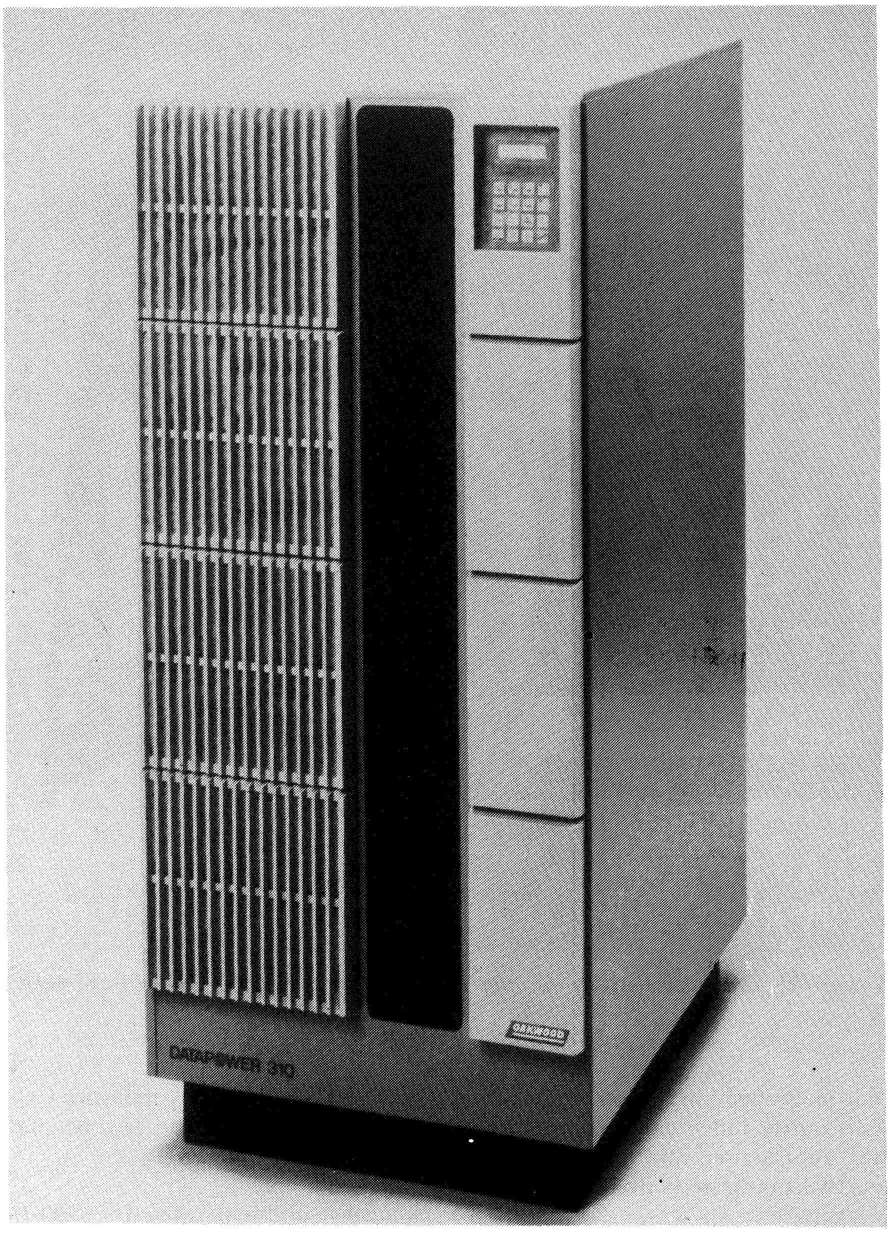

*Datapower uninterruptible power supply (UPS) systems protect computer systems, voice and data communications equipment, and plant room operations from any AC mains power supply disturbance or complete power failure.*

### FT-Raman spectroscopy accessory

An FT-Raman Accessory is now available for Perkin-Elmer's Model 1700X FT-IR Spectrometers. The FT-Raman Accessory mounts directly on to the Model 1700X, giving fluorescence free spectra so that real-world samples, such as polymers and pharmaceuticals, can be analysed. A range of optimized accessories is available for minimal sample preparation. The normal sample compartment and external beam position is left free for FT-IR analyses.

The Model 1700X FT-IR Spectrometer has a wide range beamsplitter covering the 10 000 to 370 $cm^{-1}$ frequency range, so that both FT-Raman and mid-IR spectroscopy can be performed on the same instrument.

*For further information, contact Perkin-Elmer Limited, Maxwell Road, Beaconsfield, Buckinghamshire HP9 1QA, UK. Tel.: 0494 676161; fax: 0494 678324.*

### Diode array spectrophotometers

A new series of Diode Array spectrophotometers has been introduced by Beckman. The DU 7000 Series features patented FSQ Full Spectrum Quantitation, allowing the determination, in concentration units, of the individual components in complex mixtures. Significant improvements in accuracy are achieved by using information from the entire spectrum. Typical applications include the identification, quantification and characterization of enzymes, proteins and nucleic acids, and in laboratories where samples are as small as 5 μl.

With FSQ the data are calculated using advanced Vector Quant mathematics, i.e. Fourier transforms in combination with p-matrix mathematics.

Two other features – the RediRead and RediScan modes – permit readings or wavelength scans to be taken in seconds even when another project is in progress. Measurements in progress are held, the new readings or scan set up automatically and, with the aid of a one-button prompt, the interrupted operation is resumed.

Nucleic acid concentration reporting uses Warburg and Christian coefficients, the ratio of reading at 260 and 280 nm, as well as other wavelengths of interest with background correction as requested. A general method is also included.

Protein analysis is simplified with preselected parameters for Bradford at 595 nm, Lowry (high sensitivity at 750 nm or low sensitivity at 500 nm), Biuret at 540 nm and a Direct UV Method at 280 nm.

Enzyme activity is determined by automatically calculating the rate in delta A per minute, and multiplying by any user-selectable factor.

Kinetics analyses are run at single or multiple wavelengths. Results are displayed in Michaelis-Menton, Lineweaver-Burk, Eadie-Hofstee, Hanes-Woolf or Hill Plot formats, with determinations of Km, Vmax, $k_{cat}$ and the Hill Coefficient.

*Details from Beckman, Progress Road, Sands Industrial Estate, High Wycombe, Buckinghamshire, UK. Tel.: 0494 441181.*

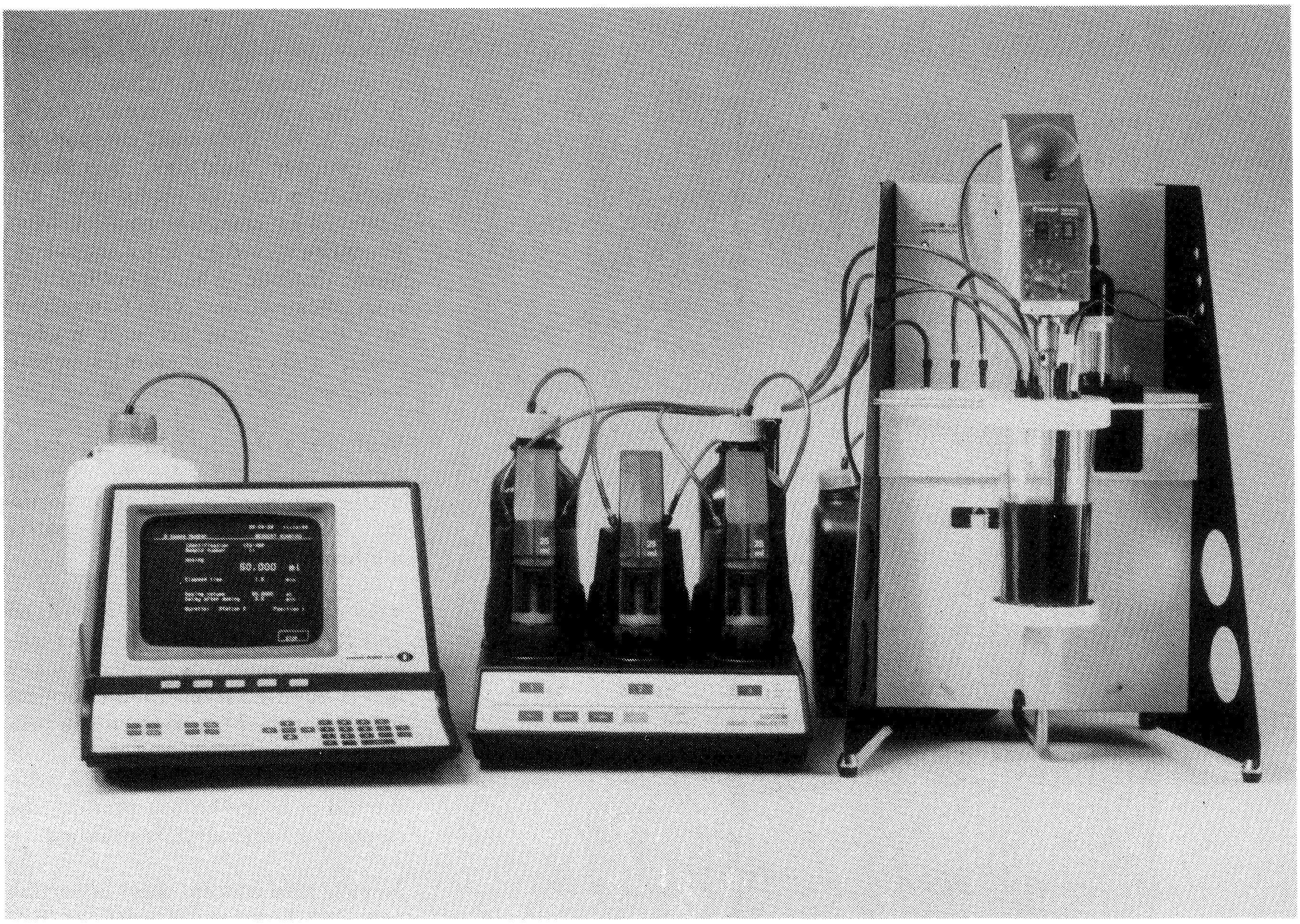

*Radiometer Analytical's titration system which simplifies and automates determination of the Kappa number for the pulp and paper industry.*

### Application software for the liquid chromatographer

Omega 235 is the latest software package from PE Nelson, for use with their OMEGA-2 and OMEGA-4 Chromatography Workstations. Designed to meet the needs of the liquid chromatographer who needs validation of his methods, it allows acquisition and manipulation of spectral data produced by the LC-235 diode array detector.

The Omega 235 package is in two parts. First, an 'add-in' application provides automatic upload of spectral data at the end of each chromatographic run. Chromatogram and spectrum, or two overlaid spectra can be displayed in split-screen mode. Second, a Spectral View program, run alongside the OMEGA software, provides comprehensive spectral manipulation facilities. These include multiple spectral overlay, plot magnification, spectral division with purity index calculation, spectral subtraction and first through fourth spectral derivatives.

*For further information, contact Perkin-Elmer Ltd, Maxwell Road, Beaconsfield, Buckinghamshire HP9 1QA, UK.*

### General-purpose thermometer

Comark Electronics offer a new thermometer which can be used by any operator for any application. Priced at £125, this new model 9003 offers exceptional value. Rugged and water resistant, portable and battery powered, the instrument can be used in the harshest industrial environment or true field use.

The 9003 is compatible with Comark's full range of Type K thermocouple probes and sensors, and is switchable to instantly measure Celsius or Fahrenheit and is dust and water resistant to IP67.

The measurement range is −100 to +1200 °C and −150 to +2000 °F with a resolution of one tenth of a degree up to +1000 °C or +1000 °F. The display indicates the battery status, if the measurement is over range, or if there is an open circuit. The configuration is retained even after the instrument is switched off.

*Details from Comark Electronics Ltd, Artex Avenue, Rustington, Littlehampton, West Sussex BN16 3LN, UK. Tel.: 0903 771911; fax: 0903 785773.*

### Kappa number determination for the paper pulp industry

The Kappa number is the most important control parameter in pulping. Radiometer Analytical A/S has manufactured titration equipment for decades, and in co-operation with the paper pulp industry, has now developed a dedicated titration system which simplifies and automates determination of the Kappa number.

As Kappa number analyses have to be performed 24 hours a day, equipment must be reliable and operation should be extremely simple. The KTS1 Kappa Number Titration System guides the day-to-day operator by two straight-forward screen pictures, one for entering sample data (for instance weight and identification) and one presenting the calculated result.

The weight may be entered directly from a balance or manually via the keyboard. An interface for connection of a printer for print-out of results and/or parameters is standard; also an external computer can be directly connected, thus providing the Kappa number result direct to the mill's process computer.

The supervisor may modify the method parameters for special purposes and implement special formulas for calculation of results. Determination of the Kappa number can also easily be implemented. All procedures are based on the TAPPI/SCAN/ISO standards. Up to 60 different variations of the Kappa/K determination can be stored.

The Kappa analysis comprises controlled stirring, addition of three reagents, and the final titration using thiosulphate as titrant. The whole procedure is based on a rigid and reproducible time schedule. With the KTS1 Kappa Number Titration System, the analysis runs smoothly and automatically.

The materials used withstand the aggressive permanganate solution, and the formation of minor amounts of $MNO_2$ does not block the liquid channels. All reagents are dosed using Radiometer precision burettes for 25 or 50 ml. The complete analysis is controlled and presented by the VIT90 Video Titrator with special software.

*For further information contact Radiometer Analytical A/S, Krogshojvej 49, DK 2880 Bagsvaerd, Denmark. Tel.: 45 31696311; fax: 45 44490011.*

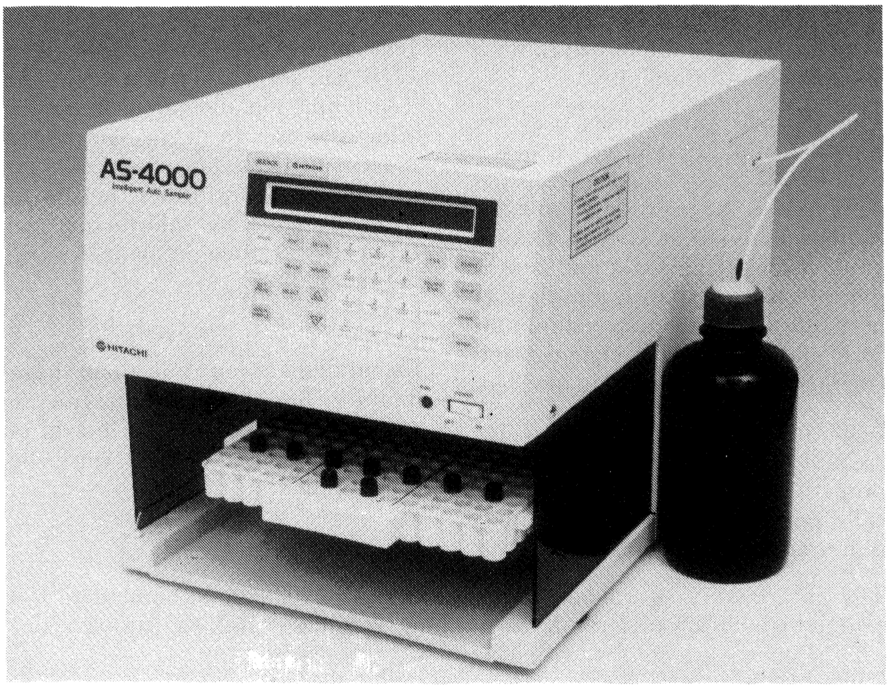

*The LiChroGraph AS 4000 Robotic Autosampler from BDH.*

### 1 μl from 1·5 μl

Chromacol's SCI-VI System of versatile micro-vials has been used recently in a case where the available sample volume was only 1·5 μl and the analyst still had to take a full 1 μl for his analysis. Using Chromacol's 03-CVG, a round-bottomed 300 μl vial, the analyst was in fact able to take 1 μl from 1·5 μl.

This is exactly the type of problem that the SCI-VI system was designed to solve, and the new 200 μl tapered vial should enhance the range's ability to work with very small volumes.

The SCI-VI system is based on the concept of two vials which, together with a range of Chromacol support sleeves, ensure compatibility with widest possible number of chromatography autosamplers.

The SCI-VI system is compatible with chromatographic autosamplers from Carlo Erba, Hewlett Packard, Perkin-Elmer, Varian and many others.

*More information from Chromacol, Glen Ross House, Summers Row, London N12 0LD, UK.*

### Colour quality

Heraeus Equipment Ltd has supplied a paper manufacturer, James Cropper, with a new Xenotest 150S light and weather fastness tester.

To ensure that its products reach the specified British and international standards, the Kendal-based paper manufacturer will use the equipment to provide an accurate assessment of the colour stability of high quality writing papers.

With a compact design, the Xenotest 150S features state-of-the-art technology including ultrasonic humidification, digital indications and control of the test conditions, including temperature and humidity. The unit is renowned for its reliability, requires little maintenance and is easy to use.

*For further product information contact Heraeus Equipment Ltd, 9 Wates Way, Brentwood, Essex CM15 9TB, UK. Tel.: 0277 231511.*

### HPLC autosamplers

The latest Merck-Hitachi Autosamplers for HPLC are now available in the UK from BDH. They are the LiChroGraph AS 2000 Autosampler and the LiChroGraph AS 4000 Robotic Autosampler.

The AS 2000 Autosampler is a very compact unit capable of accommodating 50 or 100 vial racks and designed to be a robust unit for routine analysis. It is easy to program through the LCD and repetitive injections from the same vial can be made. The injection volume for each vial can also be changed and emergency samples quickly inserted.

A special feature is that the machine recognizes when vials or racks are not properly positioned and automatically prevents damage to the injection needle.

The AS 4000 is a robotic Autosampler which can perform derivatizations, dilutions and additions. Programming is fully flexible through an LCD and special function keys. The Teach function and cursor keys even allow the user to define the precise movement of the injector needle for use with beakers, tubes, bottles or other vessels. This information can be stored to form a permanent working method.

Remarkably even injections from liquid – liquid extractions can be made. This is because the height of the injection needle can be programmed.

The AS 4000 can accommodate 150 or 200 vials and so can run unattended overnight or even over the weekend.

The AS 2000 and AS 4000 have a number of common features. In both models injection needles move rapidly in the *x-y-z* positions. Both have excellent communications facilities which include RS232C, PAN (Hitachi Personal Area network), start/stop and busy functions. Reproducibility is very good – 0·5 % CV with a 10 μl injection volume. Both units have options for thermostating vials and operate without the need for a compressed air supply.

*For further information contact the Chromatography Product Manager, BDH Ltd, PO Box 8, Dagenham, Essex RM8 1RF, UK.*

